# Clinical and Neuropathogenetic Aspects of Human African Trypanosomiasis

**DOI:** 10.3389/fimmu.2019.00039

**Published:** 2019-01-25

**Authors:** Peter G. E. Kennedy, Jean Rodgers

**Affiliations:** ^1^Institute of Infection, Immunity and Inflammation, College of Medical, Veterinary and Life Sciences, University of Glasgow, Glasgow, United Kingdom; ^2^Institute of Biodiversity, Animal Health and Comparative Medicine, College of Medical, Veterinary and Life Sciences, University of Glasgow, Glasgow, United Kingdom

**Keywords:** human African trypanosomiasis, sleeping sickness, neurology, CNS, tsetse fly, diagnostic staging

## Abstract

Trypanosomiasis has been recognized as a scourge in sub-Saharan Africa for centuries. The disease, caused by protozoan parasites of the *Trypanosoma* genus, is a major cause of mortality and morbidity in animals and man. Human African trypanosomiasis (HAT), or sleeping sickness, results from infections with *T. brucei (b.) gambiense* or *T. b. rhodesiense* with *T. b. gambiense* accounting for over 95% of infections. Historically there have been major epidemics of the infection, followed by periods of relative disease control. As a result of concerted disease surveillance and treatment programmes, implemented over the last two decades, there has been a significant reduction in the number of cases of human disease reported. However, the recent identification of asymptomatic disease carriers gives cause for some concern. The parasites evade the host immune system by switching their surface coat, comprised of variable surface glycoprotein (VSG). In addition, they have evolved a variety of strategies, including the production of serum resistance associated protein (SRA) and *T. b. gambiense*-specific glycoprotein (TgsGP) to counter host defense molecules. Infection with either disease variant results in an early haemolymphatic-stage followed by a late encephalitic-stage when the parasites migrate into the CNS. The clinical features of HAT are diverse and non-specific with early-stage symptoms common to several infections endemic within sub-Saharan Africa which may result in a delayed or mistaken diagnosis. Migration of the parasites into the CNS marks the onset of late-stage disease. Diverse neurological manifestations can develop accompanied by a neuroinflammatory response, comprised of astrocyte activation, and inflammatory cell infiltration. However, the transition between the early and late-stage is insidious and accurate disease staging, although crucial to optimize chemotherapy, remains problematic with neurological symptoms and neuroinflammatory changes recorded in early-stage infections. Further research is required to develop better diagnostic and staging techniques as well as safer more efficacious drug regimens. Clearer information is also required concerning disease pathogenesis, specifically regarding asymptomatic carriers and the mechanisms employed by the trypanosomes to facilitate progression to the CNS and precipitate late-stage disease. Without progress in these areas it may prove difficult to maintain current control over this historically episodic disease.

## Introduction

Human African trypanosomiasis (HAT), also known a sleeping sickness, is one of the world's classical “neglected diseases.” Transmitted by the bite of the blood-sucking tsetse fly of the *Glossina* genus, HAT is caused by protozoan parasites of the *Trypanosoma* genus (1–3). The incidence of HAT shows a latitudinal gradient with reported cases occurring in sub-Saharan regions between the latitudes 14° North and 29° South. It has been estimated that about 60 million people throughout 36 African countries are at risk from developing the disease, one which is usually, but not always, fatal if untreated or inadequately treated (4). There are two clinical variants of HAT, the West African form caused *by Trypanosoma brucei gambiense* (abbreviated to *T. b. gambiense*) and the East African form caused by *T. b. rhodesiense* parasites (5). The term “*brucei*” reflects the pioneering work on the disease carried out by the Scottish microbiologist Sir David Bruce in the last decade of the nineteenth century when the links between the parasite, the fly vector and wild and domestic animals were established unambiguously (5). Although *T. b. gambiense* disease is more common and causes about 95–97% of the reported HAT cases, with *T. b. rhodesiense* cases constituting the other 3–5% of reported cases, it has been estimated that the latter disease is responsible for about 18% of the total risk of infection throughout sub-Saharan Africa (6) as well as being the cause of 72% of cases that occur in European and US travelers to endemic regions of Africa for the primary purpose of visiting the African game parks (7). Though much less common and widespread, the disease caused by *T. b. rhodesiense* is a more acute and severe one compared with that due to *T. b. gambiense*.

African trypanosomiasis is a major cause of both mortality and morbidity in both man and domestic livestock and there is a close relationship between human and animal trypanosomiasis (also known as *nagana*). The main reservoir of infective trypanosomes in *T. b. gambiense* are other humans whereas domestic and wild animals such as cattle are the main reservoir of infection for HAT caused by *T. b. rhodesiense* (4) (though some animals can also harbor *T. b. gambiense*). If domestic cattle are infected with trypanosomiasis then they will be ineffective for food and milk production with significant socio-economic consequences so the animal disease and the human disease should not be considered in isolation. The problem has been compounded by the failure to reduce significantly the tsetse fly-infested regions of sub-Saharan Africa over the last 100 years, an area that continues to be in the region of a third of the land mass of the African continent (8).

## Epidemiology

Since it was first recognized HAT has been characterized by disease epidemics (especially in the early twentieth century), periods when the disease has been kept under relative control, particularly so in the 1960s, and also periodic disease resurgences which have often been associated with wars. When estimating the incidence of new HAT cases at different times there has always been the problem of under-diagnosis and/or under-reporting so one may only be detecting the “tip of the iceberg.” However, despite these caveats, it is clear that there has been a steady and indeed significant decline in new cases of HAT over the last 10–20 years. This is the consequence of massive and concerted collaborative efforts to control the disease by WHO, non-government organizations, African governments, the pharmaceutical industry, and research charities and Institutions. The key measures that have allowed this achievement have been to detect, isolate, and treat human HAT cases to prevent disease spread and also to provide more effective vector control. There was a large increase in HAT cases in the 1980s which continued for over a decade and WHO estimated in 1998 that there were about 300,000 new cases per year in Africa (5, 9). But by 2006 WHO estimated that this high figure had been reduced to 50,000–70,000 cases (4, 10). By 2009 WHO estimated that the number of new cases per year had been reduced to below 10,000 for the first time (9,878 cases), and in 2014 only 3,796 cases were reported with < 15,000 estimated cases (3, 11). By 2016 the reported number had been further reduced to 2,184 cases. In all these reports the majority of cases were due to *T. b. gambiense*, and there has been a relatively greater reduction in the number of these cases compared to *T. b. rhodesiense*. While most reported cases of HAT due to *T. b. gambiense* are to be found in the Democratic Republic of Congo (DRC), most of the reported cases of *T. b. rhodesiense* disease are found in Uganda and Malawi (3). However, in Uganda both variants of HAT have been detected (4) with the prospect of some patients being co-infected with the two diseases which raises important potential issues in diagnosis and treatment.

Interestingly, the number of documented HAT cases in the DRC in 2007 was actually slightly more than twice the number of cases reported to WHO (12), so a degree of caution should be exercised in interpreting the incidence figures. However, it is abundantly clear that these concerted control efforts have been very successful in reducing the incidence of HAT. It should also be emphasized that HAT continues to pose a serious potential risk to travelers to sub-Saharan Africa (East or West) with most cases due to *T. b. rhodesiense*, and 94 HAT cases were reported in non-endemic countries between 2000 and 2010 (7). The likely reason for this is that many European and US travelers to Africa visit the game reserves which are mainly located in East Africa, where *T. b. rhodesiense* is commonly found in wildlife reservoirs.

## Biological Considerations

Some essential features of the interactions between the trypanosome parasite and the animal or human host are given here in brief, but these have been described in detail elsewhere (3, 4). The trypanosome is a unicellular protozoan parasite, and 10% of its 9,000 genes code for variant surface glycoproteins (VSG). These proteins are distributed over the surface of the trypanosome and play a major role in determining its immune specificity. The VSG are attached to the trypanosome's outer membrane by glycosylphosphatidylinositol anchors. During infection of the host a constant low frequency gene conversion process occurs which switches the VSG genes in and out of the expression site. As a result there is a continuous process of antigenic variation which enables the trypanosome to constantly evade the host's immune responses which would otherwise have the ability to destroy the parasite. This phenomenon of antigenic variation, which is also shown by some other pathogens, explains why vaccination against the trypanosome infection has hitherto proved to be unfeasible (4, 8).

Humans have evolved innate defenses against some trypanosome species. There are proteins present in human serum which are capable of producing lysis of animal trypanosomes, called trypanolytic factors. These factors are comprised of apolipoprotein A1 (APOA1), apolipoprotein L1 (APOL1), and haptoglobin related protein (HPR) contained within two serum protein complexes, trypanosome lytic factor 1 and 2 (TLF-1 and TLF-2) (13). However, the two trypanosome species which infect humans, that is, *T. b. rhodesiense* and *T. b. gambiense*, have evolved mechanisms to overcome this lytic resistance factor. In the case of *T. b. rhodesiense* the parasite contains the *SRA* gene which encodes the serum resistance associated protein (SRA) which is able to bind to TLF, thereby conferring resistance to lysis. The mechanism of overcoming TLF-induced lysis used by *T. b. gambiense*, which lacks the *SRA* gene, is somewhat different and more complex, involving a reduction in binding affinity of the parasite receptor for TLF, the involvement of a mutated form of VSG known as *T. b. gambiense*-specific glycoprotein (TgsGP) and increased production of cysteine proteases that degrade APOL1 (13). It has also been shown recently that different *APOL1* gene variants, known as G1 and G2, which are associated with a recessive kidney diseases risk, affect an individual's susceptibility to HAT, resulting in the positive selection of certain variants in sub-Saharan Africa (14). Thus, G2 was associated with a protective effect against *T. b. rhodesiense* and a faster progression of *T. b. gambiense* disease, whereas G1 was associated with asymptomatic but infected individuals and the failure to detect trypanosomes (14).

The detailed lifecycle of the parasite has been described comprehensively elsewhere (3, 5). Starting at the point where the tsetse fly bites an infected individual or animal, the fly ingests a “blood meal” which also contains trypanosomes. When the latter enter the fly's midgut they undergo a series of biochemical, anatomical and pharmacological changes, and multiply prior to moving to the salivary glands where the now infective parasites are concentrated. The cycle is continued when the tsetse fly then bites a human or animal host. During the first, early, or “haemolymphatic stage” of infection the parasites multiply by binary fission and spread throughout the bloodstream, lymphatic system and lymph nodes, and also the systemic organs including the liver, spleen, heart, endocrine organs, and visual system (15). A chancre, which is the primary skin lesion, may or may not appear at the site of the tsetse fly bite. When the trypanosomes traverse the blood-brain barrier (BBB) to enter the central nervous system (CNS), a process which usually occurs after a few weeks in the case of *T. b. rhodesiense* and a few or several months in the case of *T. b. gambiense* disease, this marks the start of the second, late, or “encephalitic stage” of the disease. This stage is characterized by a number of neurological features (see below) and the CNS pathology shows a meningoencephalitis with a typical inflammatory infiltrate, perivascular cuffing, and cells called Morular or Mott cells which are typical of HAT and consist of plasma cells containing aberrant Ig (15). The tempo and overall duration of disease progression is different in the two disease variants, with *T. b. rhodesiense* typically causing an acute disease which, if untreated, is likely to kill the patient in a few weeks whereas the disease caused by *T. b. gambiense* is typically a more chronic disease which can last for many months and indeed sometimes even longer for years. The reasons for these pathological, and therefore clinical, differences between the two variants is not known for certain though it may possibly reflect a longstanding evolutionary adaptation of *T. b. gambiense* to the human or animal host.

## Clinical Features

The diverse clinical features of early and late- stage HAT broadly reflect the underlying pathology of the disease, except that, unlike the pathological features, there are no clinical criteria that can reliably distinguish or predict the onset of the early and late clinical stages which tend to run in to each other seamlessly (5). Although there are numerous clinical features that are typical of many cases of early and late- stage HAT, it should be appreciated that these may vary considerably. Thus, there are marked clinical phenotypic differences seen between the two variants of HAT, between travelers from non-endemic countries and patients residing in Africa, between patients in different geographical regions of one African country, and also between patients living in different African countries. Despite the relatively recent recognition of the clinical heterogeneity of HAT, it is still possible to list the commonly observed symptoms and signs.

Early-stage symptoms may be vague and non-specific which may delay the clinical suspicion and diagnosis. Thus, such features as lassitude, intermittent fever, arthralgia, and headache may initially predominate raising the possibility of malaria which may of course co-exist in patients with HAT (1). As the parasites spread within the bloodstream and lymphatic system corresponding systemic features may then follow. Such patients in endemic areas may therefore show evidence of lymphadenopathy (especially in the posterior cervical region in the case of *T. b. gambiense* disease which is known as Winterbottom's sign), haemolytic anemia, hepatomegaly and abnormal liver function, splenomegaly, endocrine disturbances, cardiac involvement such as pericarditis and congestive cardiac failure as well as ophthalmic involvement (1, 15). The clinical presentation in travelers from non-endemic countries who are infected has been reported to be somewhat different and atypical as they present with mainly gastro-intestinal symptoms such as diarrhea and jaundice in association with an acute febrile illness (16), and lymphadenopathy is rare in these latter individuals. The reason(s) for the clinical differences in the presentation of HAT in these various populations are as yet unclear and studies investigating possible genetic influences are on-going. The relatively low numbers of white travelers infected with HAT may also be a factor in accurately determining variations in disease presentation. It has been recognized recently that neurological symptoms and signs may be seen in early-stage disease which is somewhat counter-intuitive but may reflect underlying pathogenetic mechanisms. Thus, it was found that the clinical profiles of HAT may be focus-specific in that cranial nerve palsies, urinary incontinence, somnolence, tremor, and abnormal gait were detected in some patients with early stage *T. b. rhodesiense* disease in two distinct regions of Uganda (17). Further, a retrospective study of patients in a hospital in Uganda found that 26.7 and 6.7% of the early stage *T. b. rhodesiense* patients presented with late stage signs of sleep disorder and mental confusion, respectively (18). The pathogenetic significance of these interesting observations has yet to be elucidated but they do indicate the great importance of accurate and objective methods of disease staging.

Once the parasites have crossed the BBB to enter the CNS, the late or encephalitic stage of HAT is initiated with a wide variety of neurological symptoms and signs being potentially manifest. The progression and duration of the disease and the neurological disability is faster in *T. b. rhodesiense* compared to *T. b. gambiense* disease. Neurological features referable to most parts of the nervous system have been described though some are more typical. A characteristic sleep disorder with daytime somnolence and nocturnal insomnia is common in both types of HAT in endemic areas (and indeed gives the disease its name) with one large prospective study reporting its incidence in 74.4% out of a total of 2,541 patients (19). However, sleep disturbances occur only rarely in travelers with HAT (16). Initially in the late-stage there may be non-specific symptoms such as irritability, lassitude, psychiatric, and behavioral disturbances which may obscure the actual diagnosis and/or suggest alternative diagnoses. A wide variety of motor symptoms and signs may occur involving the pyramidal or extrapyramidal systems with involuntary movements including tremors, slurred speech, headache, myelopathy, myositis, and cerebellar ataxia (1). Sensory involvement is also well recognized such as sensory symptoms and motor neuropathy. A variety of visual features may also occur such as optic neuritis, diplopia and local eye complications such as iritis, keratitis, and conjunctivitis (1). If untreated the patient will deteriorate inexorably with progressive impairment of consciousness, incontinence, seizures, and eventually death in most cases.

The geographical differences in the clinical manifestations of HAT are also notable. Related to this, the severity and speed progression of late stage disease in *T. b. rhodesiense* patients can also be genetically determined in that genetically distinct parasite genotype variants have been reported as being associated with these two clinical aspects in geographically separated regions of Uganda (20). The clinical features of HAT can differ even in different regions of the same country as evidenced by a study (17) which found that the phenotype of *T. b. rhodesiense* differed in the spatially separated areas of Soroti and Tororo in the country of Uganda. In the former region neurological involvement was apparent earlier and the transition from the early to the late- stage was faster than in the latter region, though neurological dysfunction was more severe in Tororo. Phenotypic differences in *T. b. rhodesiense* cases can also vary in different countries as evidenced by a study of 138 patients with late stage disease (21) which found that typical HAT-specific neurological features were frequently observed in patients in Tanzania compared to those in Uganda where the neurological features in the late-stage were more non-specific such as fever, headache, general body pain, and joint pains.

It has been known for several years that a few patients with evidence of *T. b. gambiense* HAT infection survive and are asymptomatic without treatment. This has been confirmed in some detail over the last decade and shows that HAT is not always a fatal illness which is effectively a significant change in the established disease paradigm. This type of phenomenon is well recognized in certain breeds of African cattle such as the Ndama and West African Shorthorn which are “trypanotolerant” due their genetic ability to resist both the anemia and parasitaemia normally induced by trypanosomal infection (8). While some West African patients with *T. b. gambiense* HAT, who had declined treatment, are able to be “resistant” to infection in that they continue to be asymptomatic, aparasitaemic, and also seronegative, others are also asymptomatic and aparasitaemic following an established infection but continue to be seropositive and might be regarded as slightly different and therefore “tolerant” of infection (22–24). An intriguing and important question is just how frequent these phenomena are as the answer to this is unknown at present. It is also unclear whether such tolerant individuals represent a potential source of infection to others and whether they should be treated when detected. Furthermore, there have been reports of a few patients with *T. b. gambiense* disease who have survived for many years without treatment, and indeed a recent report described the case of a man who presented with HAT at least 29 years after infection (25) which is the longest duration of HAT infection ever reported. Of possible relevance to such observations is the recent discovery that the skin is a significant and hitherto overlooked reservoir for vector-borne African trypanosomes (26). This very important study detected the presence of extravascular trypanosomes in human skin biopsies in undiagnosed individuals who were aparasitaemic. Further, the authors showed that substantial quantities of trypanosomes existed within the skin following experimental animal (mouse) infection, which could be transmitted to the tsetse vector. These findings raise major issues not only in terms of current diagnostic methods (see below) but also for strategies that are being devised for eventual control and eradication of HAT (26).

## Neuropathogenesis of HAT

The routes and mechanisms employed by the parasite to enter and persist within the CNS remain an area of much debate. *In vitro* blood-brain barrier (BBB) models have shown that trypanosomes can cross a human brain microvascular endothelial cell layer producing only a transitory reduction in barrier integrity (27). Further studies suggest that both parasite and host enzymes, together with induction of calcium signaling pathways at the endothelial cell layer, could be fundamental in the ability of trypanosomes to circumvent the BBB and gain entry to the CNS (28, 29). The effect of trypanosome infection on BBB function has also been investigated in rodent models of the disease (30–32). Studies tracing intravenously injected fluorescent dyes in trypanosome infected rats found increasing penetration of the brain parenchyma as the infection advanced through the later stages (30) suggesting a progressive deterioration in BBB function. This dysfunction was also seen in murine infections when BBB integrity was monitored using contrast-enhanced MRI, however in this case, leakage of contrast agent was detected prior to the onset of late-stage disease and increased as the infection progressed (31, 32). This early and progressive development of BBB impairment has also been seen in human cases of *T. b. rhodesiense* infection. Using the CSF/serum albumin quotient as a measure of BBB function, a deterioration in integrity was detected in 6% of patients presenting in early-stage disease and increased to 42% of patients in the encephalitic stage (33).

**Figure 1:**
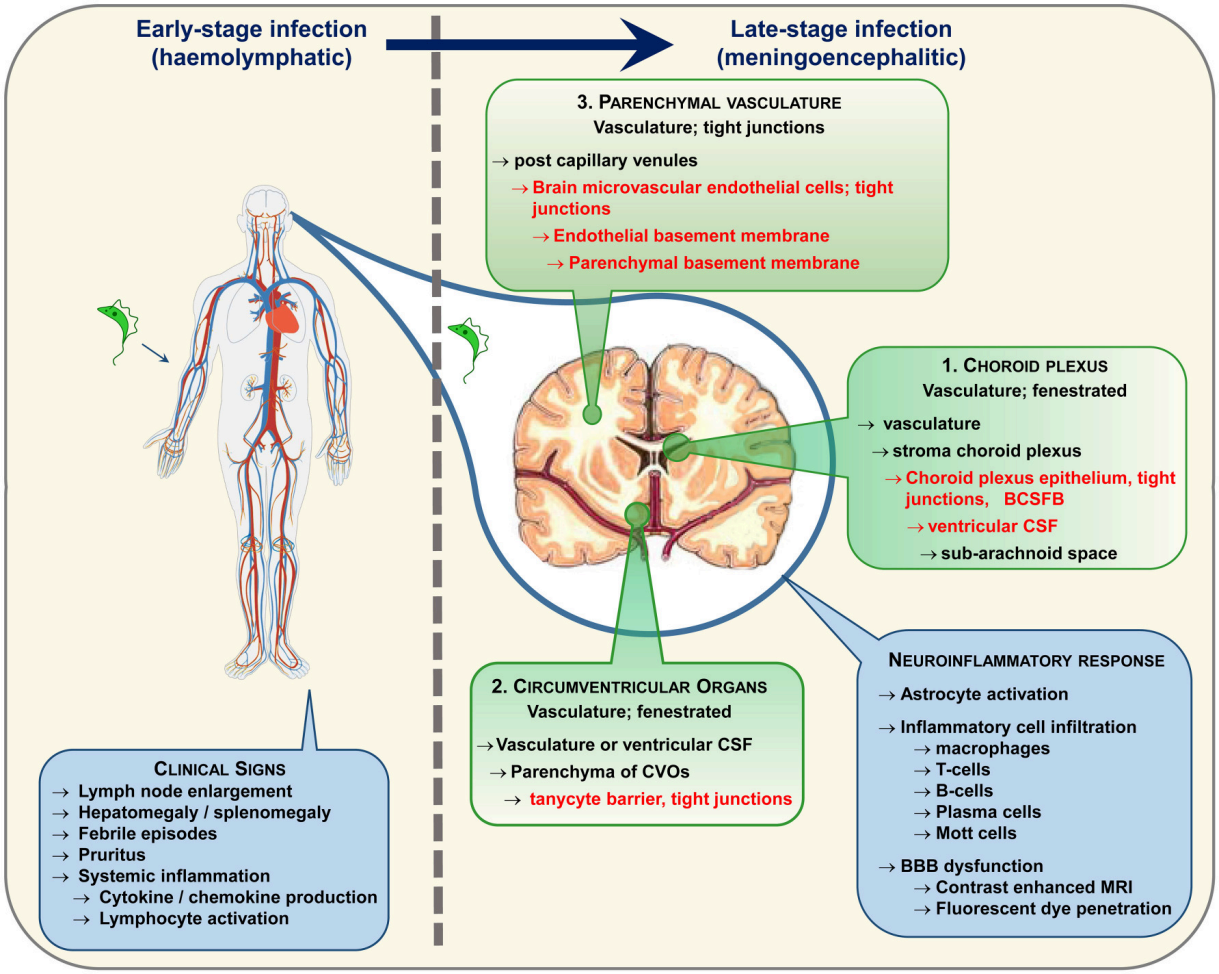
Following infection trypanosomes spread via the haemolymphatic system to invade the peripheral organs. The infection precipitates an array of non-specific symptoms mostly linked to the systemic immune reaction. When the disease progresses from the early to the late-stage the trypanosomes enter and establish within the CNS. The routes exploited by the parasites to invade the CNS remain a topic of debate. The three main possibilities are highlighted. In each case the parasites must circumvent a potential barrier (listed in red text) to gain entry to the neuropil. It is likely that a combination of these pathways is utilized. Progression to late-stage induces a series of neuroinflammatory changes in the brain characterized by a pronounced astrocyte activation and lymphocyte infiltration. As the disease advances, progressive deterioration in blood-brain barrier (BBB) function has been noted following systemic injection of fluorescent dyes and contrast enhanced magnetic resonance imaging.

Animal models of HAT have facilitated in depth investigations of disease progression and parasites have been found within the brain tissue using this controlled approach. Trypanosomes have been detected in the neuropil in both primate (34) and rodent (30, 35–38) models of late-stage infections and more recent studies, employing confocal microscopy to image labeled parasites, suggest that trypanosomes invade the CNS very quickly following infection (39, 40). Whether these parasites gain entry to the brain via the CSF or by a haematogenous route remains equivocal. Studies by Mogk et al. (41, 42) and Wolburg et al. (43) suggest that the parasites may initially cross the fenestrated vessels in the choroid plexus (Figure 1) to access the stroma before traversing the blood-CSF-barrier and disseminate with the CSF through the ventricles to the cisterna manga and sub-arachnoid space from where they can populate the pia mater. However, in these studies the parasites failed to enter the neuropil from the pia mater. Paradoxically, CSF seems to prove a hostile environment for trypanosomes. *In vitro* studies have shown that the parasites will only survive for 20 h in CSF and that survival time is further reduced if CSF from late-stage HAT patients is employed in the culture medium (44). The unfavorable nature of CSF has been purported as the cause of parasite migration from the sub-arachnoid space into the pial cell layer (42). Fenestrated vessels are also present within the circumventricular organs (Figure 1) and trypanosomes have been shown to occupy these regions in addition to the choroid plexus early after infection (45, 46). However, the circumventricular organs are isolated from other brain regions by another barrier system formed by tanycytes and this cell layer would restrict further passage of trypanosomes into the brain parenchyma (45). Recent studies have captured images of parasites entering the neuropil directly from the blood vessels (40) providing further evidence to suggest that trypanosomes have the ability to traverse the BBB *in vivo*. In human infections parasites have very rarely been detected within the brain parenchyma on post-mortem microscopic examination (47) although they are more often found within the cerebral blood vessels (47–49). This is perhaps to be expected since trypanosomes would deteriorate very quickly following death of the host and many of the cases examined had been treated with trypanocidal drugs (49, 50). Therefore, the exact route and molecular mechanisms facilitating trypanosome traversal into the brain parenchyma remain to be elucidated although a combination of all three pathways seem the most probable scenario (Figure 1).

The availability of genetically modified organisms has revealed several important factors in trypanosome-CNS invasion and the generation of the associated neuroinflammatory reaction. These include factors involved in both the innate and adaptive immune response. Innate immune response pathways involving Toll-like receptor (TLR) 2- and TLR9—MyD88 signaling can stimulate the expression of pro-inflammatory molecules such as TNF-α and IFN α/β. By utilizing a series of TLR 2^−/−^, TLR 9^−/−^, and MyD88^−/−^ mice Amin et al. demonstrated a reduction in leucocyte and parasite transmigration into the CNS in the knockout animals that appeared to correlate with reduced transcription of *TNF*-α and *IFN*-α*/*β (51). Additional inflammatory mediators including IFN-γ (52, 53) and CXCL10 (53) have been shown to play a vital role in this invasion process with trypanosomes and T-cells being confined between the endothelial and parenchymal basements membranes of the cerebral post-capillary vessels in IFN-γ^−/−^ mice (52). The laminin subtype present within the basement membrane also influences the ability of trypanosomes and T-cells to enter the CNS with regions containing laminin α4 being permissive to transmigration while areas composed of laminin α5 deterred traversal (52). Treatment of trypanosome infected mice with minocycline, an antibiotic known to reduce the passage of T-cells into the CNS in experimental allergic encephalomyelitis (EAE), provided further evidence supporting a relationship between lymphocyte crossing of the BBB and parasite brain invasion (54). In this study reductions in the number of both CD45^+^ cells and parasites were seen following drug administration together with a significant inhibition in the transcription of intercellular cell adhesion molecule-1 (ICAM-1) and E-selectin (54).

Animal models have also been essential in monitoring the effect of trypanosome infection on the expression of inflammatory mediators in the CNS, the balance of which could be crucial to the outcome of the disease. An association between high CNS concentrations of IFN-γ and TNF-α and a more severe neuroinflammatory response has been demonstrated while higher levels IL-10 and IL-6 were allied with mild CNS inflammation (55). In agreement with these findings reduced concentrations of plasma IFN-γ and TNF-α, lowered CNS parasite burden and reduced neuropathology together with an improved clinical presentation were seen following systemic IL-10 administration in trypanosome infected mice (56). The reduction in plasma IFN-γ and concomitant reduction in CNS parasitosis and neuroinflammatory reaction is an interesting finding due to the crucial role, described earlier, that INF-γ appears to have on lymphocyte and trypanosome brain invasion.

## Diagnosis of Infection and Disease Staging

It is important to establish a positive diagnosis of HAT as soon as possible after suggestive symptoms have started especially in view of the toxic nature of current drug treatment and the dangers of leaving the disease untreated. A high index of suspicion would be present if such an individual with typical symptoms is known to have been bitten by a tsetse fly in an endemic region of sub-Saharan Africa. Trypanosome parasites may be visualized on a thin or thick peripheral blood smear which is usually possible in the case of *T. b. rhodesiense* disease because of the typically high blood parasite levels in such individuals (57). By contrast, the cyclical parasitaemia with only intermittently high blood parasite levels typically seen in *T. b. gambiense* often makes direct demonstration of the parasite less likely though this should still be looked for carefully. A greater adaptation of *T. b. gambiense* to the host is thought to account for this difference so a positive diagnosis for this variant usually needs to be made by serological methods which themselves may be problematic. The Card Agglutination Test for Trypanosomiasis (CATT) has been used extensively for disease screening and is of considerable use in regions where there is a high prevalence of disease (2, 4), but it has a high incidence of false positive results which makes it particularly problematic in areas where the disease prevalence is low. A “false” positive occurs in a case that cannot be confirmed by the presence of active blood paraesitemia. The test also requires an electricity supply which may be difficult in the African field. While some advanced molecular techniques such as the Polymerase Chain Reaction (PCR) have been tested in HAT there have been significant technical issues with these and they would also not be very practical under field conditions (4).

Not surprisingly, much effort has been made recently into developing alternative and more effective tests to diagnose HAT. The development of what are now called Rapid Diagnostic Tests (RDTs) is promising to transform our ability to make this diagnosis for *T. b. gambiense* cases that require serological methods of detection. A prototype rapid diagnostic test called SD BIOLINE HAT has been developed (58) and was shown in a multi-centric prospective non-inferiority study to recognize two trypanosome antigens (VSG LiTat 1.3 and VSG LiTat 1.5), and its high sensitivity and specificity for HAT indicated its ability to become an alternative screening method to the CATT; it also does not require electrical instruments. It should be emphasized, however, that both CATT and SD BIOLINE HAT detect antibodies produced in response to identical VSG antigens, so from an immunological point of view, the two tests measure the same “event” using exactly the same immunological concept, but with a different readout. It should also be appreciated that although both assays have a very good negative predictive value, and all patients that require treatment are detected, they score relatively poorly in finding “true” active infections. For example, repeated bites by *T. b. brucei* infected flies will induce an anti-trypanosome response in the human host, but will not establish an infection, and even a bite by a *T. b. gambiense* infected fly could induce an immune response but may not necessarily result in infection. A follow-up study showed that a recombinant antigen-based RDT was as specific as, but more sensitive than, the SD BIOLINE HAT, and was as sensitive as but slightly less specific than the CATT (59). This suggested that the use of more than one screening test for HAT might be optimal. Further, it was shown recently that some RDTs that detect *T. b. gambiense* disease in humans also cross-react with antibodies found in cattle (60), findings that indicate that they may possibly be useful in some studies of animal trypanosomiasis. Demonstrating the final elimination of the disease may also be challenging when relying on diagnostic tests that are based on the detection of a host antibody response. Since the antibody response can remain viable for a considerable period after the initial antigen encounter, these test will not distinguish between patients exhibiting an active infection and those that have been successfully treated and then re-exposed to the VSG, through non-productive infected tsetse bites, thereby boosting the antibody response. This could lead to a scenario where passed exposure to HAT would be detected rather than on-going infections masking the true incidence of the disease.

Accurate and reliable methods of disease staging to distinguish the early from the late stages of HAT are extremely important both because it is not possible to stage the disease on clinical grounds alone, and also because the drug treatment for late- stage disease is so toxic (see below). However, attempts to define the late- stage on objective criteria have been controversial and problematic in some cases. The currently accepted method of identifying the late stage of both types of HAT when the CNS has been invaded is the examination of the Cerebrospinal Fluid (CSF) by lumbar puncture. The WHO criteria for defining late-stage disease is more than 5 White Blood Cells (WBC)/mm^3^ or the presence of trypanosomes in the CSF or both (9), though a higher WBC cut off point such as 20 WBC//mm^3^ is used by some physicians and investigators, especially in West Africa. A major problem may arise in the case, for example, of a patient with 6 WBC/mm^3^ in the CSF who may not in fact have CNS disease but who nevertheless using the WHO criteria would be treated with toxic late stage drugs. There is no universal consensus as to either the biological definition of late-stage disease or indeed the CSF criteria that are used to determine late stage treatment (5). Attempts to circumvent this staging problem using appropriate biomarkers of CNS disease have been made and have included, for example, levels of CSF IgM (61), neopterin (62), and combined panels of chemokines and proteins (63), but the central problem with this approach is that the results are compared with the WHO criteria which are themselves controversial and not a “gold standard.” This is the “circular argument” problem that investigators in this field have to contend with. A recent analysis of this issue has suggested a novel “reverse” approach in which statistical methods could be used to test the performance of combinations of established laboratory variables as staging biomarkers to correlate with the CSF WBC/trypanosomes and clinical features of HAT (64). It is hoped that in this way a particular CSF WBC can be identified as giving a more reliable indication of CNS involvement in HAT, thereby establishing the CSF WBC diagnosis on a firmer footing. Though non-invasive methods to identify CNS stage disease have been investigated such as polysomnography to detect alterations of sleep structure in late- stage HAT (65), and also actigraphy to measure body activity and sleep/wake cycles (66), at the current stage of development these useful diagnostic adjuncts are more likely to be used to detect disease relapses after treatment rather than serve as primary indicators to serve as a firm basis for initiating late stage treatment when it is first administered. This disease staging issue would, of course, assume less importance if an effective and non-toxic treatment for late-stage disease were to become available.

## Current Treatment of HAT

Current pharmacological therapy for HAT relies on just a few drugs all of which were developed many years ago and have a degree of toxicity which is most severe in the case of drugs for late-stage disease. This subject has been described in detail elsewhere (4, 15), and some essential aspects and also recent therapeutic developments are given here. It is only relatively recently that the pharmaceutical agency, working with non-government organizations and various academic researchers and Institutions, has focused on developing new drugs for late-stage HAT.

Drug treatment for early-stage disease for both HAT variants is effective and less toxic than that for late-stage disease (3, 4). For early-stage *T. b. gambiense* drug therapy consists of intramuscular (preferable) or intravenous or pentamidine. This drug (which can also be given as second-line treatment of *T. b. rhodesiense*) has reported side-effects that include hypotension, abnormalities of glucose metabolism, renal dysfunction, and gastro-intestinal symptoms (15). Treatment for the early-stage of *T. b. rhodesiense* is with intravenous suramin (which can also be given as second-line treatment of *T. b. gambiense*) and, though effective, has the potential side-effects of mild renal dysfunction, peripheral neuropathy, anaphylactic reactions, and bone marrow toxicity resulting in peripheral blood abnormalities (1, 5).

Drug treatment for late-stage disease in both variants is more problematic. The only treatment currently available to treat late-stage *T. b. rhodesiense* HAT is intravenous melarsoprol which was first used in 1949. Though every effective, melarsoprol is painful to administer and is very toxic, with a fatality rate of about 5–9% due in main part to a severe post-treatment reactive encephalopathy (PTRE) which develops in 5–10% of cases about half of whom die (3, 4). The actual cause of the encephalopathy is unknown but may relate to the toxic effects of massive and rapid parasite killing with a resultant cytokine “storm.” The PTRE is characterized by coma, seizures, and cerebral oedema which is usually treated with corticosteroids, anticonvulsants, and intensive support. Other complications of melarsoprol include peripheral neuropathy, skin rashes, cardiotoxicity, and agranulocytosis (4, 15). Although an abridged 10 day intravenous melarsoprol treatment schedule is now standard, the mortality rate of this regime still remains in the region of 5.9% (67). Though also effective against late-stage *T. b. gambiense*, melarsoprol is now second line therapy for this variant. First-line therapy for *T. b. gambiense* is now NECT (nifurtimox-eflornithine combination therapy) which is a combined course of intravenous eflornithine (DFMO) (an ornithine decarboxylase inhibitor) and oral nifurtimox (68). NECT is less toxic than melarsoprol and is equally if not more effective and for several years has been the initial therapy of choice for this variant. However, NECT is not effective against *T. b. rhodesiense*. While eflornithine monotherapy has also been given for *T. b. gambiense* this is more toxic and less well tolerated than NECT (3), so is not usually given. Potential side-effects of eflornithine include alopecia, bone marrow toxicity, gastro-intestinal features, and seizures. Whether drug resistance to eflornithine may develop in the future is possible but remains to be seen.

There are several new drugs for late-stage HAT in the pipeline, in development or under evaluation. A drug of the nitroheterocyclic group called fexinidazole is the focus of much current interest. A recently published study reported that in a phase 2/3 randomized non-inferiority trial of oral fexinidazole vs. NECT therapy, in late-stage *T. b. gambiense* HAT in the DRC and Central African Republic, oral fexinidazole successfully treated 91% of patients at the 18 month assessment point compared with a 98% treatment success rate with NECT, and the treatment-related side effects were similar in both groups (69). This is clearly a significant advance though the efficacy of fexinidazole was less than that of NECT, and it remains to be seen whether this drug is also effective in late-stage *T. b. rhodesiense* disease. An effective oral drug has a number of advantages, but at the current time both of the current authors (PGK and JR) would still opt for NECT as first-line therapy in the unfortunate event that they developed late-stage HAT. Another promising drug currently under evaluation is a new oral compound of the oxaborole group. This drug, called SCYX-7158 cures CNS-stage HAT in an experimental mouse model (70), was safe in a phase 1 trial, and is currently undergoing evaluation in a phase 2/3 trial. Another very different approach has been to develop a form of melarsoprol that can be given orally but which retains its ability to be effective against HAT while losing its toxicity. Complexed melarsorpol is formed by inserting melarsoprol inside a cyclodextrin molecule to form a fusion drug consisting of cyclodextrin-melarsoprol inclusion complexes (71). This drug cured trypanosome infections when given orally in a reproducible mouse model of HAT and was free of toxic effects. The drug has dual European Medicines Agency (EMA)/US Food and Drug Administration (FDA) approval as an orphan drug for African trypanosomiasis and the EMA scientific protocol advisory committee has approved a protocol for a phase 2 trial in *T. b. rhodesiense* HAT and it is hoped that such a trial will be conducted when appropriate funding becomes available.

## Prospects for Eventual Control of HAT

While it would clearly be ideal to eliminate HAT completely from the whole of sub-Saharan Africa, the more realistic goals of WHO are to eliminate sleeping sickness as a public health problem by 2020 and to achieve interruption of its transmission by 2030. These goals could be achievable, though there are several challenges and caveats that should be taken into account.

HAT has frequently showed its ability to resurge even after it had appeared to have been brought under control as was the case, noted above, in the 1960s. This indicates that it may prove to be a formidable task to realize both the WHO goals in time. Related to this, it will be necessary for all the relevant Institutions and agencies concerned to keep a very careful watch over the changing prevalence of HAT and never to relax the constant monitoring of infected individuals so they can be treated promptly. Some cases may reside in remote regions of sub-Saharan Africa which poses a problem for effective screening and may cause underestimates of infection to be made. War poses a particular problem in this regard because of the inevitable breakdown of social structures and difficulties in identifying infected individuals. The problem may be compounded by the difficulties in identifying patients in the early stage of disease because the early symptoms are frequently non-specific. Further, the fact that cattle are the animal reservoirs of infection for *T. b. rhodesiense* disease will make it very difficult, and perhaps impossible, to achieve complete control of this HAT variant. Mass culling of domestic and wild animals is not a realistic proposition, but the fact that this variant only causes a minority of HAT cases, despite its severity and propensity to affect Western travelers, may possibly mitigate in part this issue. The prospects for eventual control of animal trypanosomiasis will also be limited, and the control and even elimination of the tsetse fly vector for both the animal and human disease in all but a few regions also seems unlikely especially when one considers how little of the areas infested by the tsetse fly have diminished over the last 100 years. Another significant issue is that the true frequency of “asymptomatic” infected individuals is not currently known. If such individuals are not identified by mass screening they could potentially be a source of infection with *T. b. gambiense* HAT, and this could hamper control efforts. Further, the recent identification of the skin and extravascular tissues in asymptomatic humans as a reservoir of trypanosomes could also impair efforts to achieve the elimination of HAT as a public health problem, especially if such individuals prove to be a source of infection to other people. There is also no guarantee that current drugs will be able to reach and kill the parasites in these previously unrecognized areas of the body. Finally, there remains the underlying concern that drug resistance may develop against both currently used and future drugs especially if they are given by the oral route. While it is hoped that the WHO aims are achieved within the intended time frame, at least for *T. b. gambiense* HAT, it is clear that there will be several significant obstacles that will need to be overcome.

## Author Contributions

All authors listed have made a substantial, direct and intellectual contribution to the work, and approved it for publication.

### Conflict of Interest Statement

The authors declare that the research was conducted in the absence of any commercial or financial relationships that could be construed as a potential conflict of interest.
